# Differential effects of antidepressant treatment on long-range and short-range functional connectivity strength in patients with major depressive disorder

**DOI:** 10.1038/s41598-017-10575-9

**Published:** 2017-08-31

**Authors:** Jing An, Li Wang, Ke Li, Yawei Zeng, Yunai Su, Zhen Jin, Xin Yu, Tianmei Si

**Affiliations:** 1 Peking University Sixth Hospital/Peking University Institute of Mental Health/Key Laboratory of Mental Health, Ministry of Health (Peking University)/National Clinical Research Center for Mental Disorders (Peking University Sixth Hospital), 100191 Beijing, China; 2 Department of Radiology, 306 Hospital of People’s Liberation Army, Beijing, China

## Abstract

Although we have some basic understanding of the neurochemical mechanisms of the antidepressants, the network-level effect of antidepressant treatment is still not fully understood. This study was conducted to investigate the effects of antidepressant on functional brain networks of patients with major depressive disorder (MDD). We performed resting-state fMRI scans on 20 first-episode drug-naive MDD patients at baseline and after escitalopram medication for 8 weeks. Twenty healthy controls also received MRI scans with an 8-week interval. The graph theory indices, long- and short-range functional connectivity strength (FCS), were computed to characterize the brain connectivity. The analysis of covariance was conducted on FCS maps of patients and controls to obtain the interaction effect of group and time, which indicate treatment-related effect. Following treatment, increased long-range FCS in the bilateral posterior cingulate cortex/precuneus and right thalamus in MDD patients at baseline were reduced. Meanwhile, increased short-range FCS in the bilateral ventromedial prefrontal cortex and left amygdala in patients were reduced, while reduced short-range FCS in the right parahippocampal gyrus was increased. Results suggest that the brain regions associated with negative emotional processing and regulation, and self-referential function could be modulated by escitalopram treatment; long- and short-range FCS are differentially affected by antidepressant.

## Introduction

Major depressive disorder (MDD) is a globally prevalent disorder with multi-dimensional clinical features involving emotion, cognition, and somatic domains. MDD has been conceptualized as a brain disorder that affects information exchange across large-scale neural systems^1, 2^. Antidepressant drugs are commonly used treatments for MDD. However, the network-level mechanisms via which the neurochemical changes induced by antidepressants could translate into clinically meaningful effects is still not fully understood. Investigating the network-level effects of antidepressants may help to find more promising biomarkers for treatment evaluation of MDD.

Resting-state functional magnetic resonance imaging (R-fMRI), which examines the temporal correlation of spontaneous fluctuation of the blood-oxygen-level dependent (BOLD) signals across brain regions, provides a powerful tool to investigate the whole-brain functional network^3–5^. There is considerable evidence from the R-fMRI studies that MDD was abnormal in functional connectivity (FC) within several neural systems involving default mode network (DMN)^6–9^, salience network^9^, cognitive control network^9^, as well as cortical-limbic emotional regulation circuit^10, 11^. A recent meta-analysis showed anomalous resting-state FC among the frontoparietal network, dorsal attention network, DMN, affective network, and ventral attention network in MDD^1^. Further, intervention studies showed that antidepressant medication could partly reserve the abnormalities of intrinsic FC of MDD patients. For instance, 8-week treatment with sertraline could exert a therapeutic effect on MDD patients by strengthening the cortical-limbic connectivity^12, 13^. Escitalopram treatment additionally increased the amplitude of low-frequency fluctuation in the prefrontal cortex (PFC) and cingulate gyrus^14^. Another study showed that duloxetine treatment for 8 weeks decreased connectivity in the components of anterior DMN with the right dorsolateral, right superior frontal premotor cortex, and left inferior frontal gyrus, but increased the connectivity in the components of the DMN with the pregenual and subgenual cingulate cortex, right hippocampus, and middle occipital gyrus^15^.

The brain is composed of complex biological systems that integrate signals from multiple brain regions to ensure effective information processing^16, 17^. Graph theory provides a powerful means to quantitatively investigate the topological organization of brain connectivity. A number of topological properties, including small-worldness, network hubs, and modularity, have been characterized in the human brain^18^. Functional connectivity strength (FCS) is a voxel-wise data-driven approach for identifying the functional hubs and connectivity patterns of brain networks^19^. The voxels with higher FCS values indicate larger numbers of functional connections with other voxels, suggesting stronger ability of information transfer^19, 20^. Abnormal FCS has been revealed in MDD patients, such as reduced FCS in the bilateral middle cingulate cortex and increased FCS in the right occipital cortex^21^.

Physical distance has a potential influence on the spatial distribution of brain connectivity^22, 23^. The balance between long- and short-range connectivity is important for efficient information processing^22^. Functional brain hubs with higher long-range FCS (lFCS) are mainly located in the posterior cingulate cortex (PCC)/precuneus, medial and lateral PFC, insula, and lateral temporal and parietal cortices. Meanwhile, short-range FCS (sFCS) are prominently located in the medial PFC, lateral PFC, insula, sensorimotor, and visual cortices^23^. The lFCS integrates information among distinct modules and hubs^24^, and operates at a higher time and metabolic cost^23, 25^, whereas the sFCS reflects the local efficiency of brain activity and operates at a lower cost^22, 23, 25^. A few of studies have suggested that the brain regions showing FCS alterations in MDD patients varied with the anatomical distance of brain connectivity. Specifically, reduced sFCS in the right orbitofrontal cortex, bilateral superior temporal gyrus, bilateral precuneus, insula, right inferior parietal lobule, and occipital regions were observed in MDD patients^26, 27^. In this context, it may be important to consider the potential effect of connectivity distance in the investigation of neural mechanism of MDD.

In previous report, we have investigated the effect of escitalopram treatment on FCS in MDD patients. We found abnormally increased FCS in the bilateral dorsomedial PFC and reduced FCS in the hippocampus in MDD patients at baseline, which was reversed after 8-weeks treatment; the FCS reduction in the dorsomedial PFC was significantly correlated with symptomatic improvement^28^. However, it remains unclear whether the effects of antidepressants on brain connectivity are dependent on anatomical distance. For this reason, we conducted this work to examine the changes in the lFCS and sFCS in first-episode, drug-naive patients with MDD following escitalopram treatment for 8 weeks. We hypothesized that the clinical improvement of MDD patients would be associated with the normalization of the lFCS and sFCS in brain regions across the medial PFC and limbic system, which are important for emotional processing and regulation, and self-reflection^29^.

## Results

### Sample Characteristics

There were no significant differences in age, gender distribution, and educational level between MDD patients and HCs (Table 1). The HRSD total score decreased significantly in MDD patients after treatment for 8 weeks (ps < 0.001).

**Table 1:** Demographic and clinical characteristics of the subjects.

	MDD patients baseline (n = 20)	Healthy controls baseline (n = 20)	MDD patients week 8 (n = 20)	Healthy controls week 8 (n = 20)	p
Male/Female	9/11	9/11	9/11	9/11	—
Age (years)	34.6 ± 12.2	33.3 ± 10.3	34.6 ± 12.2	33.3 ± 10.3	0.717^a^
Education (years)	12.9 ± 2.1	13.7 ± 3.1	12.9 ± 2.1	13.7 ± 3.1	0.348^a^
Illness duration (months)	5.4 ± 6.3	—	7.4 ± 6.3	—	—
Total HRSD score	27.9 ± 4.0	0.9 ± 0.8	7.1 ± 4.5	0.9 ± 0.7	<0.001^b^/0.84^c^
Average dose of escitalopram (mg)			18.5 ± 2.9		

Abbreviation: MDD, major depressive disorder; HRSD, Hamilton Rating Scale for Depression.

^a^Indicate the P values for the comparisons between the MDD patients at baseline and the healthy controls at baseline.

^b^Indicate the P values for the comparisons between the MDD patients at baseline and the patients after 8 weeks of treatment.

^c^Indicate the P values for the comparisons between the healthy controls at baseline and the healthy controls after 8 weeks.

### FCS Patterns

**Figure 1:**
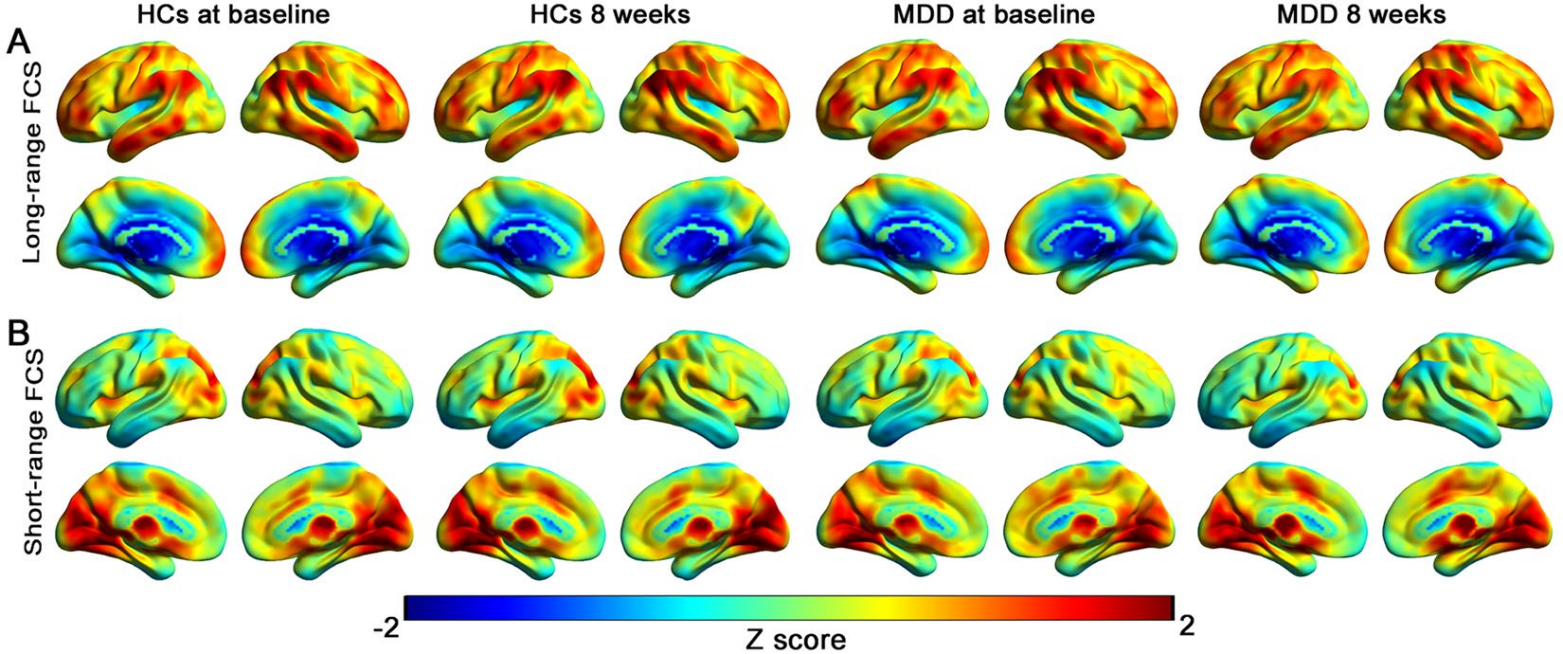
Long-range and short-range FCS patterns. (**A**) Mean long-range FCS maps in the healthy control (HC) and MDD groups at baseline and week 8. (**B**) Mean short-range FCS maps in the HC and MDD groups at the two time points. The color bars represent the connectivity strength.

As shown in Fig. 1, the spatial distributions of the FCS patterns were largely similar across the MDD patient and HC groups at baseline and week 8. The brain regions showing high lFCS and sFCS are located in the DMN, including the medial PFC and PCC. Regions showing a preferentially high lFCS but relatively low sFCS were located in the lateral parietal and temporal cortices, while regions displaying a preferentially high sFCS but low lFCS involved the visual occipital cortices and subcortical areas.

### Interaction Effects of Group and Time

**Figure 2:**
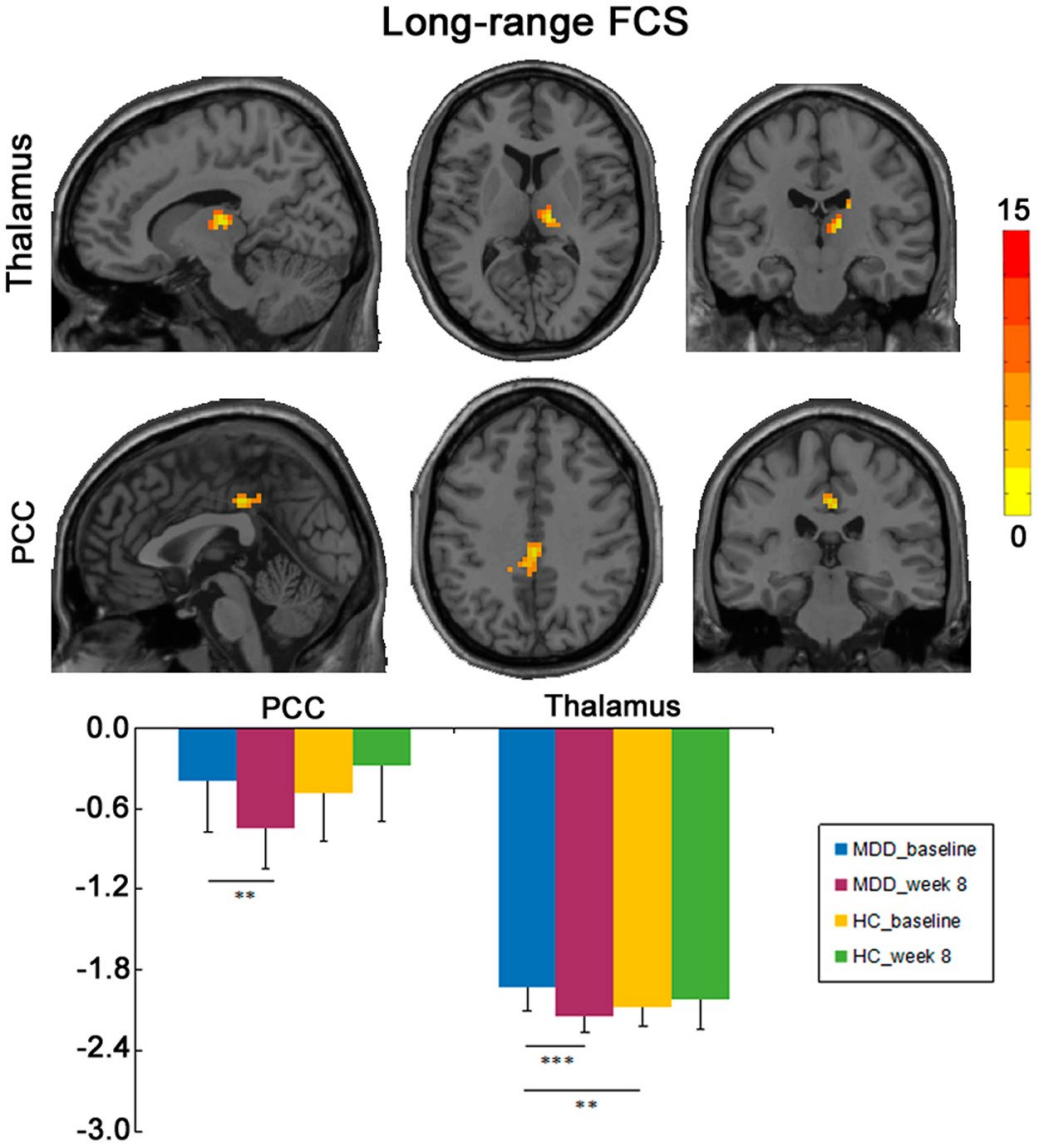
Group × time interaction on long-range FCS. (**A**) The imaging map presents the brain regions showing significant group × time interaction on long-range FCS, including the bilateral posterior cingulate cortex (PCC/precuneus) and the right thalamus. The Gaussian random field theory was used for multiple comparison correction with a cluster threshold of p < 0.05 and z > 2.3. (**B**) The bar maps showing between-groups and within-group differences in the clusters showing significant group × time interaction on long-range FCS. The data were expressed as mean value + SD. **p < 0.01, ***p < 0.001.

**Figure 3:**
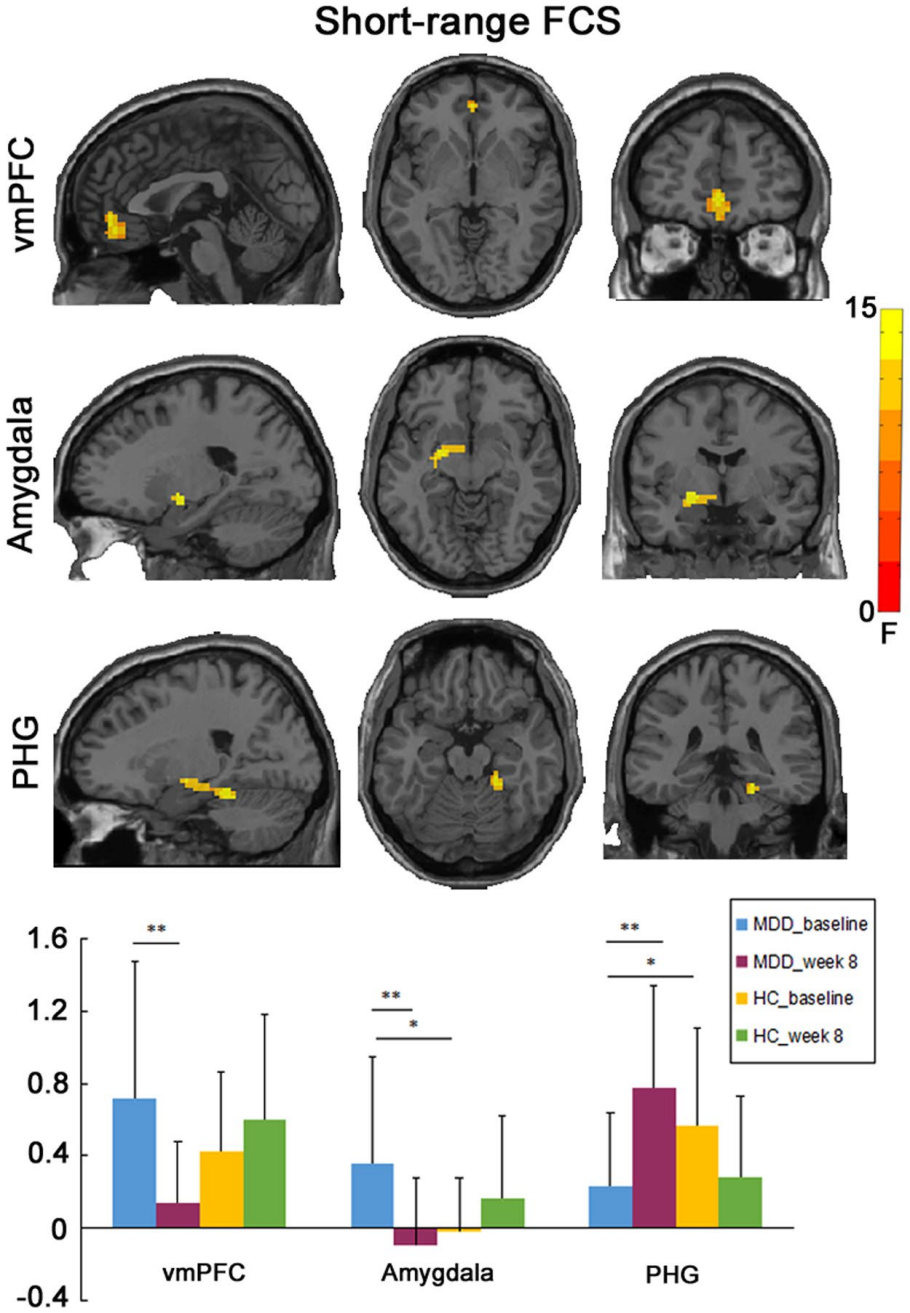
Group × time interaction on short-range FCS. (**A**) The imaging map presents the regions showing significant group × time interaction on short-range FCS, including the bilateral ventromedial prefrontal cortex (vmPFC), left amygdala, and right parahippocampal gyrus (PHG). The Gaussian random field theory was used for multiple comparison correction with a cluster threshold of p < 0.05 and z > 2.3. (**B**) The bar maps showing between-groups and within-group differences in the clusters showing significant group × time interaction on short-range FCS. The data were expressed as mean value + SD. *p < 0.05, **p < 0.01.

A significant interaction effect of group and time on the lFCS was observed in the bilateral PCC/precuneus and right thalamus (Fig. 2; Table 2). Post-hoc analysis showed that increased lFCS in the bilateral PCC/precuneus and right thalamus in MDD patients at baseline was reduced following treatment (Table 2). With respect to sFCS, a significant interaction effect of group and time was found in the bilateral ventromedial PFC (vmPFC), left amygdala and right parahippocampal gyrus (PHG) (Fig. 3; Table 2). Post-hoc analysis showed that increased sFCS in bilateral vmPFC and left amygdala in MDD patients at baseline was reduced following treatment, while reduced sFCS observed in the right PHG in MDD patients was increased following treatment (Table 2).

**Table 2:** Anatomical locations of the group × time interaction effect on FCS.

Region	Cluster size (voxels)	MNI coordinates (x, y, z)	Statistics (F)	Patients vs controls at baseline (t, p)	Patients week 8 vs baseline (t, p)
**Long-range FCS**					
Bilateral posterior cingulate cortex	45	0, −27, 39	18.76	0.72, 0.476	−4.17, 0.001
Right dorsal medial thalamus	48	12, −18, 9	15.07	3.14, 0.003	−4.38, <0.001
**Short-range FCS**					
Bilateral ventromedial prefrontal cortex	63	0, 48, −3	11.73	1.51, 0.141	−3.87, 0.001
Left amygdala	49	−21, −3, −9	13.96	2.68, 0.011	−3.61, 0.002
Right parahippocampal gyrus	72	21, −36, −18	12.73	−2.24, 0.031	3.27, 0.004

### FCS changes and clinical improvement

**Figure 4:**
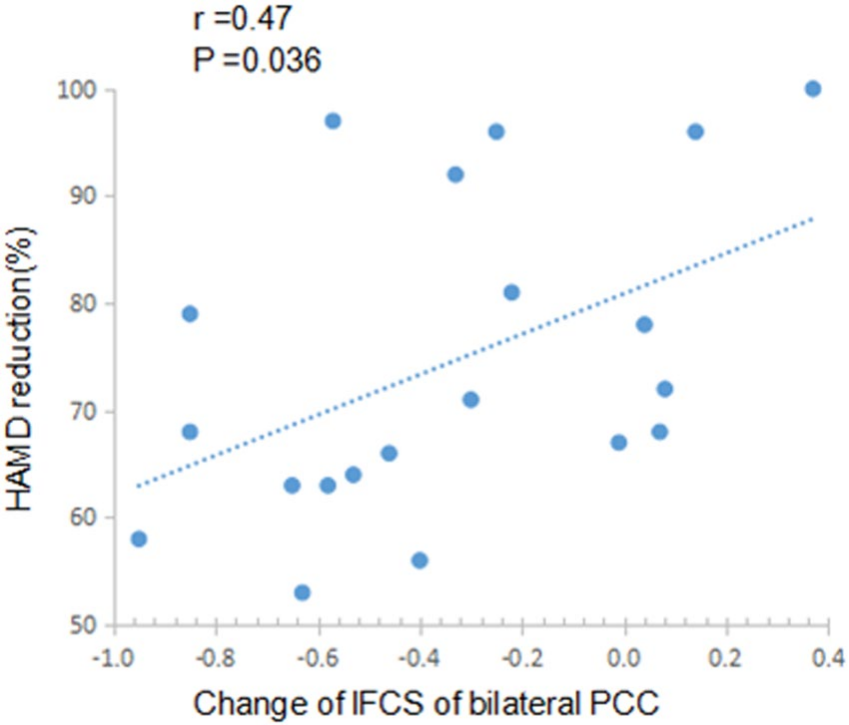
Correlation between change of lFCS of bilateral PCC and change in depressive severity. The scatter map shows significant correlation of the changes of lFCS of bilateral PCC with reduction rate of HRSD scores (HRSD_0w_ − HRSD_8w_/HRSD_0w_*100%) from week 0 to week 8. lFCS: long-range functional connectivity strength; PCC: posterior cingulate cortex; HRSD: Hamilton Rating Scale for Depression.

The mean values of lFCS and sFCS in the clusters showing significant interaction effects of group and time were extracted and then used to examine any correlations with clinical variables. The results showed significant positive correlations in the reduction of the lFCS of the bilateral PCC/precuneus with the reduction rate of HRSD (HRSD_0w_ − HRSD_8w_/HRSD_0w_ * 100%) (Fig. 4).

## Discussion

To our knowledge, this is the first study to investigate the effect of antidepressants on distance-dependent resting-state FC (indexed by lFCS and sFCS) in MDD. We found significant treatment effects on the lFCS and sFCS of MDD patients. Specifically, abnormal lFCS and sFCS in the cotical (PCC/precuneus, vmPFC) and limbic (thalamus, amygdala, PHG) regions in patients were normalized after treatment. These treatment-related regions was not been observed in our previous study^28^ in the same group of subjects, suggesting that the distance-dependent FCS is valuable for the investigation of neural mechanism of antidepressant treatment.

We found a significant effect of escitalopram treatment on the FCS in cortical midline structures, including the bilateral PCC/precuneus and bilateral vmPFC. DMN (including PCC, PFC) shows very high activation at rest and is considered as a nexus of connectivity that integrates information from various neural systems^23^. The PCC integrates the information from multiple domain-specific systems with a limited number of long-distance connections^24, 30^ and acts as a mediator for self-related cognitive processes such as episodic memory^31, 32^. Here, we found increased lFCS in the PCC/precuneus in MDD patients at baseline, which was inhibited by escitalopram treatment. These findings were compatible with previous studies, which showed a reversal of increased resting-state FC^33^ and nodal centrality^34^ in the PCC/precuneus in MDD patients after antidepressant treatment. In this context, it is plausible to propose that increased lFCS in the PCC/precuneus reflects an over-recruitment of large-scale brain networks, which may be associated with excessive memory for negative information seen in MDD patients. Down-regulating the connectivity strength of the PCC/precuneus may be potential pathway for MDD treatment. Further, a positive correlation was observed between reduction in lFCS of the PCC/precuneus and the improvement of depressive symptoms (assessed as reduction rate of HRSD scores). Based on the physiological significance of the PCC/precuneus, we propose that inhibiting the PCC/precuneus hyperconnectivity may contribute to normalized collaboration of multiple neural networks, which is important for the improvements of clinical symptoms of MDD.

Connecting with subcortical regions, the vmPFC is involved in automatic emotional regulation and self-referential activity, such as rumination^35^. Compelling evidence has highlighted MDD-related alterations in the vmPFC, with decreased gray matter volume^29^ and cortical thickness^36^, increased activity during the rest^6^ and negative self-referential emotional processing^37^ was observed in MDD patients. Excessive vmPFC-amygdala coupling has been thought to contribute to negative affect, MDD onset in vulnerable populations, and persistent experience of negative emotional distress in MDD patients^38^. This region appears critical for clinical response to antidepressant treatment, as its decreased activity was observed in MDD patients following different kinds of treatments, such as antidepressant medication^39^, placebo^40^, ECT^41^, cognitive behavioral therapy^39^, as well as repetitive transcranial magnetic stimulation (rTMS)^42^. Combined with these previous reports, the current study indicates an inhibitory effect of escitalopram on the increased sFCS in the vmPFC of MDD patients, which indicates that antidepressant may inhibit the excessive self-referential thoughts by decreasing the connectivity strength of the vmPFC.

We also observed significant changes in the FCS of the limbic system regions, including the reduction of sFCS in the left amygdala and the lFCS in the bilateral thalamus, and the increase of the sFCS in right PHG in MDD patients following treatment. The amygdala generates emotional reactions to external stimuli, whereas the thalamus is responsible for the distribution of afferent signals and considered as a “relay” that forwards signals from subcortical areas to cerebral cortex^4, 43^. The cognitive model of MDD proposes that excessive amygdala reactivity to external stimuli was associated with the generation of negative emotion^44^, which was supported by convergent evidence that among MDD patients, the amygdala was exaggeratedly activated under negative affective stimuli^14, 44, 45^. The signals of negative emotion are then routed by the thalamus to emotional regulation cortical regions^46^. Antidepressant medication, especially SSRIs, might act on the limbic regions directly by inhibiting exaggerated activity of the amygdala, thalamus, and other limbic regions in MDD patients when receiving negative emotional stimuli^15, 44, 47, 48^. Moreover, SSRI treatment (escitalopram) inhibited the hyperactivity of the amygdala and thalamus in healthy group in response to negative stimuli even after 7-day antidepressant medication^49^. Antidepressants may also act by up-regulating the FC of cortico-limbic mood-regulation circuit, as increased connectivity between the amygdala and the anterior cingulate cortex^12^, dorsal PFC^50^ was observed in MDD patients after SSRI treatment, while decreased FC in the amygdala with the precuneus and posterior cingulate cortex was found after SSRI treatment^50^. In this context, reduced sFCS in the amygdala of MDD patients following treatment observed in this study may lead to an alleviation of excessive negative processing, which may mitigate the information transmission load for negative emotion between the thalamus and cortical brain areas. Together with our findings of the FCS reduction in the amygdala and thalamus after treatment, we propose that escitalopram treatment may inhibit the signals transmission for negative emotion along the cognitive hierarchy.

The hippocampus refers to the emotional memory^51^ and is important for adaptive control of the stress through negative feedback inhibition^52^. Previous studies have shown reduced hippocampal volume^29, 51^ and low hippocampal activation in response to happy faces in MDD patients^45^, as well as reduced negative FC between the hippocampus and inferior parietal cortex, cerebellum^53^, dorsomedial PFC^54^. More and more evidence highlights the role of the hippocampus in the successful treatment of MDD, as increased hippocampal responses to happy faces after escitalopram treatment^55^, increased hippocampal volume^56^ and FC^57^ after ECT treatment were observed in MDD patients. In our current work, we found that escitalopram could normalize reduced sFCS in the right PHG, which indicates antidepressant may enhance the information communication between PHG and nearby brain regions; such an change may be helpful for strengthening the regulation of stress response and emotional memory in MDD patients.

Several issues need to be further addressed. First, as an open-label design, the absence of untreated or placebo-treated control groups make it difficult to determine whether the changes in FCS were due to pharmacological effect or other combined factors. However, there may be some potential ethical problems to recruit a group of patients who are currently experiencing severe depressive episodes and make them untreated or be given placebo for 8 weeks. Further studies could recruit patients with mild or moderate depressive episode, and placebo, other antidepressant drugs or psychotherapy could be used to help to clarify whether the brain function changes are induced by certain antidepressant treatment or other factors. Second, it is hard for our study to investigate the neural differences between treatment responders and nonresponders. Future studies could try to include treatment non-responders to seek some neural differences between treatment responder and non-responders. Last, there may be some changes before clinical improvement, future researches could collect the fMRI data at the early stage of the treatment to examine the acting mechanisms of antidepressant drug and predictors of treatment response.

The current study demonstrated that antidepressant treatment with escitalopram was associated with changes in resting-state distance-dependent FCS in MDD patients. Reversed abnormal FCS within the cortical-limbic circuit in MDD patients after escitalopram treatment may be important for the alleviation of excessive negative emotion and self-referential thoughts. This study thus provides new evidence for the understanding of neural mechanism of antidepressant treatment.

## Methods

### Subjects

Thirty-six first-episode drug-naive patients with MDD were recruited from the Peking University Institute of Mental Health and the Beijing Anding Hospital of Capital Medical University between May 2010 and January 2013. The patients were diagnosed by a psychiatrist using the Mini-International Neuropsychiatric Interview (MINI)^58^, a short structured diagnostic interview for Diagnostic and Statistical Manual of Mental Disorders, 4th fourth edition (DSM-IV). Inclusion criteria included an acute depressive episode, total score of the 17-item Hamilton Rating Scale for Depression (HRSD)^59^ ≥18, and the illness duration ≤24 months. Patients comorbiding with any other Axis I disorder, Axis II personality disorder or mental retardation in recent one year were excluded.

Among the initial 36 patients, one had excessive head movement during scanning; one reported serious adverse effects after 2 days of medication; two patients stopped medication during the study period for some personal reasons; five patients refused to participate in the second MRI scans; and seven patients changed to other antidepressant drugs due to a poor response to escitalopram during the study period. Finally, 20 patients completed the whole study. Twenty age (±5 years), gender, and education (±5 years) matched healthy control (HC) subjects were recruited from the local community. All HCs had a HRSD score less than 7 at baseline and 8-week follow-up. HCs with a HRSD score of more than 7, any current or lifetime psychiatric disorders, or a history of major psychiatric or neurological illness in their first-degree relatives were excluded.

Exclusion criteria for all subjects included serious medical or neurological illness, a history of significant head trauma, substance dependence or abuse within the last year, current or previous use of psychotropic drugs, a history of electroconvulsive therapy (ECT), acutely suicidal or homicidal, current pregnancy or breastfeeding, or any contraindications to a MRI scan. All the subjects were right-handed as determined by the Edinburgh Handedness Scale. Written informed consent forms and experimental protocol were approved by the Ethics Committee of the Sixth Hospital (Institute of Mental Health) of Peking University, and we obtained written informed consent forms from all the subjects before the study. The demographic and clinical characteristics of the subjects were provided in Table 1.

### Antidepressant Treatment

Patients were treated with oral escitalopram at 5 or 10 mg/day, then was increased to 10–20 mg/day within 7 days, and continued at this dose until they finished the 8-week study in accordance with the ethical principles originating in the Declaration of Helsinki and the International Conference on Harmonization Good Clinical Practice guidelines. The dose adjustment was accorded to the clinical judgment of the psychiatrist and the patient’s consent. The final doses of escitalopram were 20 mg/day for 15 patients, 15 mg/day for 4 patients, 10 mg/day for one patient. All of the 20 patients were given clinical assessment at baseline and the end of the 8th week. They all showed a clinical response to escitalopram treatment, defined as a reduction of 50% decrease from the baseline HRSD score; eleven of them (55%) achieved clinical remission with a HRSD-17 score of 7 or below. All patients received escitalopram without any other psychotropic medication or psychotherapies.

### MRI Data Acquisition

MRI scans were performed with a 3.0-T imaging system (Siemens Magnetom Trio; Siemens Medical Solutions, Erlangen, Germany). The resting-state functional images were collected with a gradient-echo echo-planar imaging sequence with the following parameters: repetition time (TR) msec/echo time (TE) msec, 2000/30; flip angle, 90; matrix, 64 × 64; and field of view, 210 × 210 mm^2^; thickness/gap, 4.0 mm/0.8 mm; 30 slices covering the whole brain. The total acquisition time was 7 minutes. After the functional imaging scans, a high-spatial-resolution T1-weighted magnetization-prepared rapidly acquired gradient-echo (MPRAGE) sequence was acquired sagittally (TR msec/TE msec, 2300/3.01; matrix, 256 × 256; spatial resolution, 1 × 1 × 1 mm^3^; flip angle, 9°; thickness, 1 mm; 176 slices) to achieve better registration.

For the resting-state functional scans, subjects were instructed to keep their eyes closed, remain still without head movement, not think of anything in particular, and not fall into sleep during the scan. Patients underwent the fMRI scans before and after treatment for 8 weeks. The HCs were scanned with the same procedures. The R-fMRI scan was carried out in accordance with relevant guidelines and regulations.

### Data analysis

#### Image Preprocessing

The R-fMRI images were preprocessed with Data Processing Assistant for Resting-State fMRI (DPARSF, http://rfmri.org/DPARSF)^60^, which is based on Statistical Parametric Mapping (SPM12, http://www.fil.ion.ucl.ac.uk/spm). After removal of the first ten volumes, the remaining 200 volumes were corrected for different signal acquisition times. The functional volumes were motion-corrected using a six-parameter rigid-body transformation. Then, several nuisance signals including the six head motion parameters, global mean signal, signals from the cerebrospinal fluid and white matter, and linear detrend were regressed out. Derived images were normalized to Montreal Neurological Institute (MNI) space (3 mm^3^ isotropic). Then, the transformed images were band-pass filtered (0.01–0.1 Hz) to reduce the effects of low-frequency drift and high-frequency noise.

#### Distance-dependent FCS

The FCS analysis was performed as follows. We first computed Pearson’s correlations between the time series of all pairs of voxels, constructing a whole-brain connectivity matrix for each subject. This computation was constrained within a gray matter (GM) mask that was generated by setting a threshold of 0.2 on the mean map of all GM maps involving all subjects. To improve normality, the individual correlation matrices were transformed to z-score matrices using a Fisher r-to-z transformation.

The lFCS and sFCS were calculated according to the procedures described previously^23^. The lFCS was derived as the average correlation between a given voxel and voxels to which the Euclidean range (approximately equivalent to physical distances) was more than 75 millimeter from the given voxel, while the sFCS was derived as the average correlation between a given voxel and other voxels that were less than 75 millimeter from the given voxel. Considering the ambiguous interpretation of negative correlations after removal of the global signal, we conservatively restricted our analysis to positive correlations above a threshold of r = 0.2; such a threshold was chosen to eliminate the voxels with weak correlations attributable to signal noise. The FCS maps were further smoothed with a 6 mm Gaussian kernel.

### Statistical analysis

We performed a two-way repeated-measures analysis of variance (ANCOVA), with the group (patient and control groups) used as a between-subject factor, and time (baseline and 8th weekend) used as a repeated measure. The significant interaction effect of group and time was used to indicate the treatment effect on lFCS and sFCS. The variables including age, gender, educational level were used as covariates. We used Gaussian random field theory to correct for multiple comparisons with a cluster threshold of p < 0.05 and z > 2.3. Following the ANCOVA test, post-hoc t-tests were performed on the clusters showing significant interaction effects of group and time. Specifically, the mean values of the FCS in these clusters were extracted from the patient and HC groups at two time points and were compared by two-sample t-tests between groups.

Pearson correlation analysis was performed to examine the correlations between changes in the mean values of FCS in clusters showing significant interaction effect of group and time and reduction rate of HRSD scores following treatment. The statistical significance was set at p < 0.05, uncorrected for multiple comparisons.

### Data availability

Corresponding author, Tianmei Si, owns the data, and the data can be available.
